# Bilateral intercostal, subscapular and teres major heterotopic ossifications in a 63-year-old male with COVID-19

**DOI:** 10.1093/omcr/omac024

**Published:** 2022-03-16

**Authors:** Alejandra Micolich Vergara, Salvatore Marsico, Alberto Solano López, Flavio Zuccarino

**Affiliations:** Radiology, Hospital del Mar, Barcelona, Spain; Radiology, Hospital del Mar, Barcelona, Spain; Radiology, Hospital del Mar, Barcelona, Spain; Radiology, Hospital del Mar, Barcelona, Spain; Cardiovascular Radiology, Hospital Sant Joan de Déu, Barcelona, Spain

A 63-year-old male with a history of obesity and hypertension, presented to the emergency department with fever and progressive shortness of breath.

Upon arrival he was in hypoxemic respiratory failure, the chest X-ray showed bilateral patchy ground glass opacities and a RT-PCR test (reverse transcription polymerase chain reaction) confirmed coronavirus disease of 2019 (COVID-19) pneumonia.

He developed an acute respiratory distress syndrome and was admitted to the intensive care unit where he spent 134 days hospitalized, requiring invasive mechanical ventilation.

Initial thoracic-CECT (contrast-enhanced computed tomography) scan revealed diffuse involvement of pulmonary parenchyma with ground-glass opacities, consolidations and areas of crazy-paving pattern.

**Figure 1:**
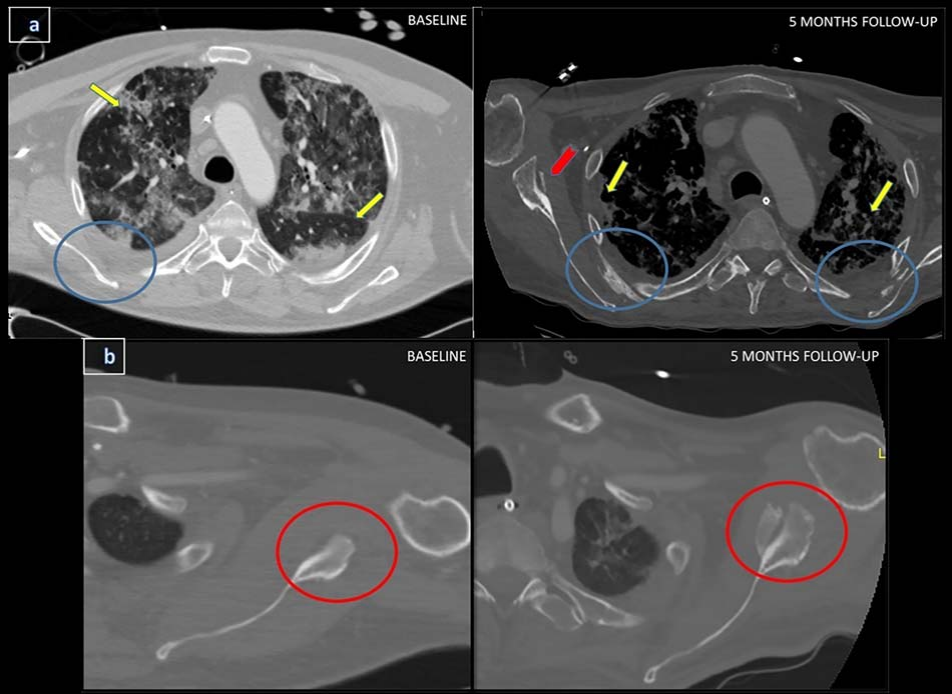
(**a**) Baseline axial thoracic-CECT images at aortic arch level and at 5 months follow-up axial thoracic-CECT images at the same level. Yellow arrows indicate the pulmonary findings related to COVID-19 pneumonia, represented mainly by bilateral ground glass opacities and peripheral lung consolidations. At the posterior chest wall region in baseline CT (left blue circles), we can confirm the absence of hyperdensity areas whereas in the follow-up CT, it clearly displays hyperdense linear images at the perimuscular soft tissue adjacent to the subscapular and intercostal muscles compatible with HO (right blue circles). In addition, at 5 months follow-up CT we can see a hyperdense linear image located in the perimuscular area adjacent to the right teres major muscle (red arrow). (**b**) Axial thoracic-CECT images at the left glenohumeral joint level and at 5 months follow-up axial thoracic-CECT images at the same level. Baseline CT shows the region adjacent to the teres major muscle (left red circle) without any involvement and in the control CT, it clearly shows hyperdense large nodular images of the perimuscular soft tissue adjacent to the teres major muscle (right red circle).

A follow-up thoracic-CECT, performed 5 months after admission because of the absence of respiratory improvement, depicted the appearance of multiple bilateral hyperdense linear and nodular images located in the soft tissue around the intercostal, subscapular and teres major muscles (Fig. 1a and 1b).

These radiological findings are highly suggestive of heterotopic ossifications (HO).

Given the patient’s severe clinical context, a histopathological confirmation was not feasible.

There are few cases described in the literature of chest wall myositis ossificans (MO; [1–3]) and, recently, two case reports of periarticular HO in COVID-19 patients have been described [4, 5].

The term MO is widely used but somehow inappropriate because the ossification is not originated within the striated muscle fibers but from the interstitial connective tissue, so the most suitable terminology is HO [1, 4, 5].

The most frequent presentation of HO is secondary to musculoskeletal trauma, surgery or burns but it can also develop spontaneously in relation to prolonged immobilization within the setting of critical care units [4, 5, 6].

To our knowledge, this is the first case of bilateral intercostal, subscapular and teres major HO in a COVID-19 hospitalized patient.

Further investigation is needed to reveal the real incidence of this condition and to understand the relationship between COVID-19 and HO development.
